# *Phaseolus vulgaris* extract ameliorates high-fat diet-induced colonic barrier dysfunction and inflammation in mice by regulating peroxisome proliferator-activated receptor expression and butyrate levels

**DOI:** 10.3389/fphar.2022.930832

**Published:** 2022-08-11

**Authors:** Carmen Avagliano, Carmen De Caro, Mariarosaria Cuozzo, Fabrizio Maria Liguori, Giovanna La Rana, Laura Micheli, Lorenzo Di Cesare Mannelli, Carla Ghelardini, Orlando Paciello, Roberto Russo

**Affiliations:** ^1^ Department of Pharmacy, University of Naples “Federico II”, Naples, Italy; ^2^ Department of Health Sciences, School of Medicine, University of Catanzaro “Magna Graecia”, Catanzaro, Italy; ^3^ Department of Neuroscience, Psychology, Drug Research and Child Health–Neurofarba-Pharmacology and Toxicology Section, University of Florence, Florence, Italy; ^4^ Department of Veterinary Medicine and Animal Production, University of Naples “Federico II”, Naples, Italy

**Keywords:** gut–liver axis, inflammation, phaseolamin, butyrate, peroxisome proliferator-activated receptor, intestinal barrier integrity

## Abstract

Obesity is a health concern worldwide, and its onset is multifactorial. In addition to metabolic syndrome, a high-fat diet induces many deleterious downstream effects, such as chronic systemic inflammation, a loss of gut barrier integrity, and gut microbial dysbiosis, with a reduction of many butyrate-producing bacteria. These conditions can be ameliorated by increasing legumes in the daily diet. White and kidney beans (*Phaseolus vulgaris* L.) and their non-nutritive bioactive component phaseolamin were demonstrated to mitigate several pathological features related to a metabolic syndrome-like condition. The aim of the present study was to investigate the molecular pathways involved in the protective effects on the intestinal and liver environment of a chronic oral treatment with *P. vulgaris* extract (PHAS) on a murine model of the high-fat diet. Results show that PHAS treatment has an anti-inflammatory effect on the liver, colon, and cecum. This protective effect was mediated by peroxisome proliferator-activated receptor (PPAR)-α and γ. Moreover, we also observed that repeated PHAS treatment was able to restore tight junctions’ expression and protective factors of colon and cecum integrity disrupted in HFD mice. This improvement was correlated with a significant increase of butyrate levels in serum and fecal samples compared to the HFD group. These data underline that prolonged treatment with PHAS significantly reduces some pathological features related to the metabolic syndrome-like condition, such as inflammation and intestinal barrier disruption; therefore, PHAS could be a valid tool to be associated with the therapeutic strategy.

## Introduction

Obesity is an abnormal or excessive fat accumulation that may impair health. In the last years, its prevalence has tripled, and it is the fifth leading cause of death globally (World Health Organization, 2021). High-fat diet (HFD) causes obesity and metabolic disorders and its prolonged consumption causes dysbiosis (Cani et al., 2007). The Western-style diet is high in fat and simple carbohydrates and low in fibers (Sonnenburg et al., 2016). Recently, it has been shown that an increase of legumes in the daily diet can improve weight control in obese patients (Ricci et al., 2014). The α-amylase inhibitor isoform 1, also called phaseolamin, is extracted from common white kidney beans (*Phaseolus vulgaris* L.), and it is well known to interfere with the breakdown of carbohydrates, reducing starch digestion and absorption (Champ, 2002). As previously reported, *P. vulgaris* is not the only source of phaseolamin but it is also widely considered safe (Chokshi, 2006; Obiro et al., 2008). *Phaseolus vulgaris* L. extract containing alpha-amylase inhibitor and phytohaemagglutinin had alleviating effects on metabolic syndrome and anti-obesity activity (Viguiliouk et al., 2017). Furthermore, we recently demonstrated that a prolonged treatment with a standardized extract of *P. vulgaris* (PHAS), containing phaseolamin, significantly reduced several pathological features related to a metabolic syndrome-like condition induced in mice by HFD (Micheli et al., 2019).

During the last decade, it was reported that among all the factors that influence the composition of the gut microbiota diet is probably the most significant (Sandhu et al., 2017). It was shown that a classic Western diet significantly reduces the level of butyrate-producing bacteria (Russell et al., 2011), and a shift to a diet rich in fibers and prebiotics can restore these bacteria abundances (Cani et al., 2008). Different evidence has shown how, through gut microbiota, diet influences stress, behavior, and cognition but above all obesity and metabolic disorders, and so nutrition is already a complementary and alternative approach (Johnson et al., 2016). Moreover, the intestinal microbiota also communicates with other organ systems including the brain, lungs, skin, and liver, influencing their function in newly discovered ways and highlighting the possible contributions of gastrointestinal dysbiosis to other bodily conditions (Kamada et al., 2013).

HFD intake leads to a detrimental modification at the intestinal level, such as a loss of gut barrier integrity, and to a low-grade inflammation throughout the body, termed “meta-inflammation”. It is a chronic state of inflammation mediated by macrophages located within the colon, liver, muscle, and adipose tissue (Li et al., 2018). Peroxisome proliferator-activated receptor (PPAR)-α and γ are members of the nuclear receptor family that regulate not only hepatic but also systemic inflammation (Bensinger and Tontonoz, 2008; Agarwal et al., 2017).

The aim of the present study was to investigate the molecular pathways involved in the protective effects on the intestinal and liver environment of a chronic treatment with a standardized seed extract of *P. vulgaris* (PHAS) on a murine model of HFD.

## Materials and methods

### *In vivo* experimental procedures

Male C57 BL/6 mice (Envigo, Varese, Italy) weighing approximately 20 g at the beginning of the experimental procedure were used. Twelve mice were housed per cage, kept at 23.0°C ± 1.0°C with a 12 h light–dark cycle. During acclimatization, they were fed a standard laboratory diet and tap water *ad libitum*. All animal manipulations were carried out according to the Directive 2010/63/EU of the European Parliament and of the European Union Council (22 September 2010) on the protection of animals used for scientific purposes. The ethical policy of the University of Florence complies with the Guide for the Care and Use of Laboratory Animals of the US National Institutes of Health (NIH Publication no. 85–23, revised 1996; University of Florence assurance number: A5278-01). Formal approval to conduct the experiments described was obtained from the Animal Subjects Review Board of the University of Florence. Control animals were fed *ad libitum* for 19 weeks with a standard chow diet (STD), with 24% protein, 58% carbohydrate, and 18% fat as a percentage of total Kcal (Envigo, Varese, Italy). Metabolic syndrome was induced by feeding the animals with a high-fat diet (HFD; Research Diets, New Brunswick, NJ) for 19 weeks *ad libitum*. The HFD diet contained 60% fat, 20% protein, and 20% carbohydrate as a percentage of total Kcal (Watanabe et al., 2012). The model is consistent with what was previously published (Micheli et al., 2019). Briefly, mice were randomly divided into three groups, ensuring no differences in body weight mean (*n* = 12 animals for each group) as follows: (1) a control group receiving a chow diet and vehicle per os (STD); (2) an HFD group receiving vehicle; and (3) an HFD group receiving daily PHAS (500 mg/kg, Indena S.p.A). For the administration, PHAS was suspended in 1% carboxymethylcellulose sodium salt (CMC; Sigma-Aldrich, Milan, Italy) and daily *per os* administered 30 min before the dark phase of the circadian light–dark cycle in the animal facility from week 11 (the time by which obesity was full-blown) until week 19. During the treatment, body weight and food intake were monitored weekly. Body weight, food intake, glucose and insulin tolerance test, HDL, LDL, triglycerides, total cholesterol, and glucose levels, together with liver damage by Hematoxylin-Eosin, steatosis index, and oxidative alteration by lipid peroxidation (TBARs), were evaluated and published in our previous work (Micheli et al., 2019).

The doses of PHAS were chosen on the basis of the literature (Micheli et al., 2019). On week 19, mice were sacrificed by cervical dislocation, and a tissue explant was performed.

### *Phaseolus vulgaris* extract preparation

The vegetal extract used (Beanblock®; Indena S.p.A., Milan, Italy) is a standardized dry extract containing an alpha-amylase inhibitor and phytohemagglutinin. Briefly, *P. vulgaris* dry extract was prepared by means of aqueous extraction and alcoholic precipitation from the common kidney bean. Bean extract was obtained by extraction with citrate buffer and precipitation with ethanol. The extract is characterized by a standardized composition of 8.5% (w/w) tested alpha-amylase inhibitor, with inhibiting activity of 1400 U/mg, and phytohemagglutinin (hemagglutinating activity of 16 hemagglutinating units/mg). The manufacturing process is described in detail by Fantini et al. (2009) and Loi et al. (2013).

### Protein extraction and western blot analysis

Colon, cecum, and liver samples were homogenized on ice-cold lysis buffer [20 mM Tris–HCl (pH 7.5), 10 mM NaF, 150 mM NaCl, 1% Nonidet P-40, 1 mM phenylmethylsulfonyl fluoride, 1 mM Na_3_VO_4_, and leupeptin and trypsin inhibitor 10 μg/ml]. After 1 h, tissue lysates were obtained by centrifugation at 12,000 rpm for 20 min at 4°C. Protein concentration was estimated by the Bio-Rad protein assay (Bio-Rad Laboratories, Hercules, CA, United States), using bovine serum albumin as a standard. Colon (40 μg), cecum (40 μg), and liver (30 μg) lysate were dissolved in Laemmli sample buffer, boiled for 5 min and separated on SDS-polyacrylamide gel electrophoresis and transferred to nitrocellulose membrane The filter was probed with anti-inducible nitric oxide synthase (iNOS) antibody (dilution 1:1000; cat. No. 610432, BD Bioscience, from Becton Dickinson, Buccinasco, Italy) or anti-cyclooxygenase (COX)-2 (dilution 1:1000; cat. No. 610204, BD Bioscience) or anti-nuclear factor κB p65 (NF- κB) (dilution 1:500; cat. No. sc-8008, Santa Cruz Biotechnology, Dallas, TX, United States) or anti-inhibitor factor κB alpha (IκB-α) (dilution 1:500; cat. No. sc-1643, Santa Cruz Biotechnology) or anti-peroxisome proliferator-activated receptor (PPAR)-α (dilution 1:1000; cat. No. P0369, Sigma-Aldrich, Milan, Italy) or anti-PPAR-γ (dilution 1:1000; cat.no. MA5-14889, Invitrogen, Rockford, IL, United States) or anti-occludin (dilution 1:500; cat. No. sc-133256, Santa Cruz Biotechnology) or anti-zonulin 1 (ZO-1) (dilution 1:1000; cat. No. 40–2300, Invitrogen, Milan, Italy) in 1 × PBS, 3% non-fat dried milk, and 0.1% Tween 20 at 4 °C overnight. The secondary antibody was incubated for 1 h at room temperature. Subsequently, the blot was developed using enhanced chemiluminescence detection reagents (Amersham Pharmacia Biotech, Piscataway, NJ, United States), according to the manufacturer’s instructions. The detection of the filter was performed by the ChemiDoc Imaging System (Bio-Rad Laboratories). To ascertain, the blots were loaded with equal amounts of protein lysates, and they were also incubated in the presence of the antibody against the β-actin (cat. no. A5441, Sigma-Aldrich) or GAPDH (cat. no. G9545, Sigma-Aldrich).

### Histological analysis

At week 19, mice were sacrificed and tissues were collected. Samples of cecum and colon were fixed in 10% formalin and embedded in paraffin, using standard procedure. Sections (5-micron) were mounted on slides and stained with hematoxylin and eosin and analyzed in a blinded manner by two independent observers for the evaluation of the histopathological score. The histological features valuated were goblet cell hyperplasia, epithelial hyperplasia, and dysplasia. The severity of goblet cell hyperplasia was graded, based on the ratio between goblet cells and total epithelial cells, into the following categories according to a score of 0–3 (0 = none; 1 = mild; 2 = moderate; 3 = severe). The ratio was obtained by counting, in a blinded fashion, total epithelial cells and cells with goblet morphology from ten high-powered fields of colon and cecum cross-sections. Similarly, the level of epithelial hyperplasia, defined as an increase in epithelial cell numbers relative to baseline epithelial cell numbers per crypt, was scored into the following categories: 0 = absent; 1 = mild; 2 = moderate; and 3 = severe. Dysplasia was scored in the following three categories: 0 = absent; 1 = mild to moderate cytologic atypia and mild architectural disturbance; and 2 = architectural abnormality and severe cytologic atypia. Dysplasia and hyperplasia distribution were both separately scored into the following categories: 0 = absent; 1 = focal; 2 = multifocal; and 3 = diffused. The grade was obtained through the evaluation of the total area of the cecum and colon cross-section.

### Fecal and serum butyrate extraction

Samples were obtained at week 19 (end of PHAS treatment); 0.5 g of fecal samples were weighed and suspended in 1 ml of pure water and vortexed. The supernatant was filtered (0.45 μM) and acidified with 20 μl of H_3_PO_4_ 85% (w/v) (Sigma-Aldrich), and vortexed for 5 min. For butyrate extraction, anhydrous diethyl ether (Sigma-Aldrich) was added to the acidified fecal homogenate samples (1:1, v/v), vortexed, and centrifuged for 30 min at 12,000 g at room temperature. The diethyl ether layer (containing butyrate) was transferred to a new glass tube containing sodium sulfate anhydrous to remove the residual water. Finally, the organic phase was placed in a new glass tube for gas chromatography–mass spectrometry (GC-MS) analysis. A standard curve (1–200 ug/ml) (butyric acid, cat. no. 19215, Sigma-Aldrich) was generated at the beginning of the run.

Serum samples were acidified with 20 μl of H_3_PO_4_ 85% (w/v) (Sigma-Aldrich), vortexed for 5 min, and incubated on ice for 5 min. The acidified samples were extracted by adding ethyl acetate (1:1, v/v), vortexed for 5 min, and then centrifuged for 20 min at 12,000 g at room temperature. Finally, the organic extract (containing butyrate) was carefully removed and transferred into a new glass tube for GC-MS analysis. A standard curve (1–50 ug/ml) was generated at the beginning of the run.

### GC/MS analysis

The GC column was an Agilent DB-WAX Ultra Inert with a length of 30 m, an internal diameter of 0.25 mm, and a film thickness of 0.25 μM. The GC was programmed to achieve the following run parameters: the initial column temperature was set at 90°C, hold of 2 min, and then increased to 100°C at a rate of 2°C/min, hold of 10 min, and finally ramp of 5°C/min up to a final temperature of 110°C for a total run time of 21 min, gas flow of 70 ml min^−1^ splitless to maintain 12.67 p.s.i. column head pressure, and septum purge of 2.0 ml min^−1^. Helium was the carrier gas (1.5 ml min^−1^ constant). Parameters of mass spectrometer were source at 230°C and MS Quad at 150 °C. A blank solvent (ethyl acetate) was injected between every sample to ensure no memory effects.

### Statistical analysis

Data from Western blots were expressed in an arbitrary unit of OD ratio of β-actin. Statistical analysis was performed by the analysis of variance (ANOVA) test for multiple comparisons followed by Bonferroni’s post hoc test, using GraphPad Prism (GraphPad Software, San Diego, CA, United States). Differences among groups were considered significant at values of *p* < 0.05.

For the histological assay, statistical analysis was performed by the analysis of variance (ANOVA) test for multiple comparisons followed by Bonferroni’s test, using SPSS Statistic software 24.0 (IBM, New York, NY, United States). The same statistical software was used for the correlation analysis between PHAS anti-inflammatory activity and PPAR expression.

## Results

### *Phaseolus vulgaris* extract treatment mitigates liver pro-inflammatory enzyme expression and restores peroxisome proliferator-activated receptor expression

**FIGURE 1:**
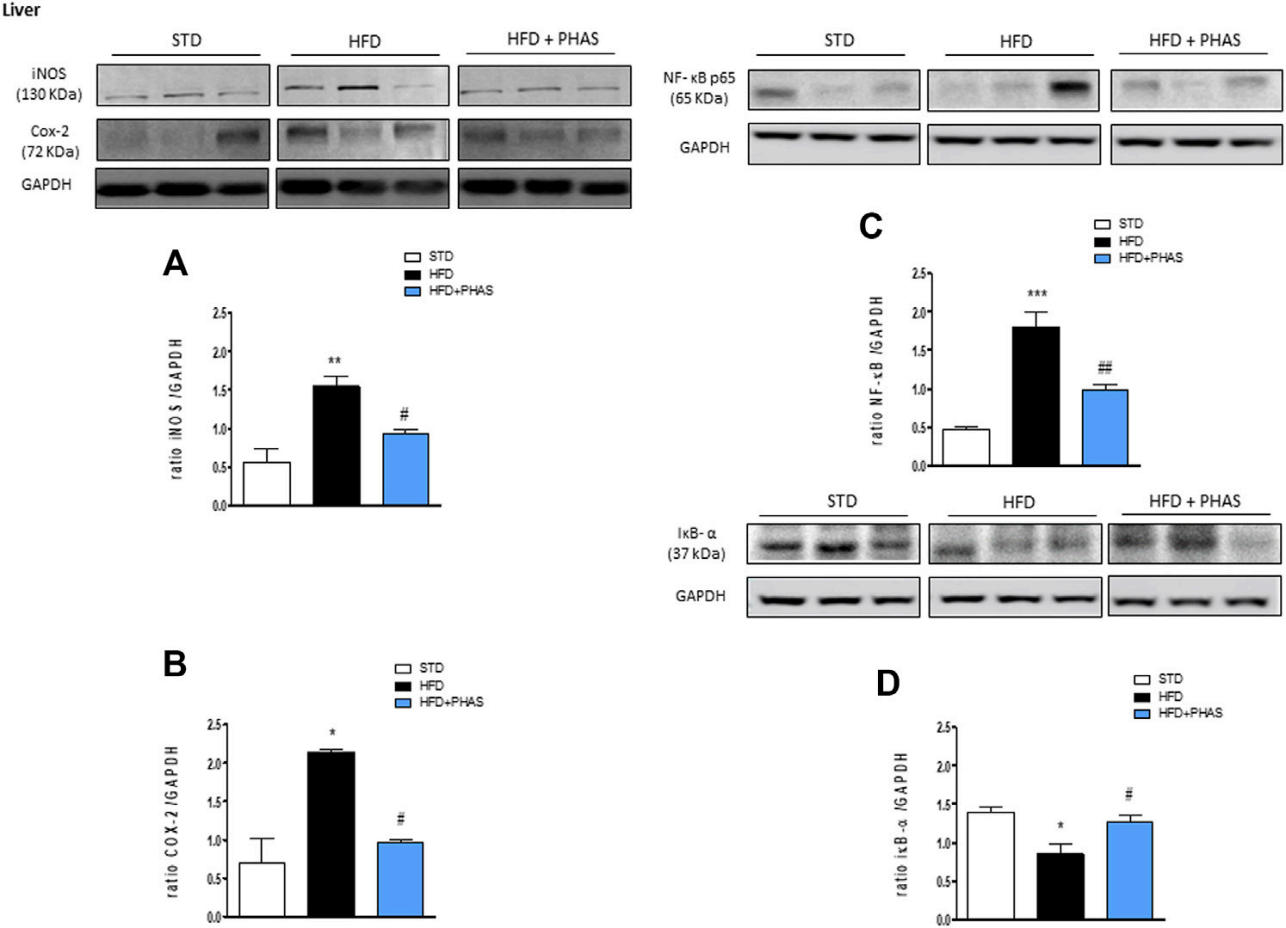
Anti-inflammatory effect of *P. vulgaris* extract (500 mg/kg, PHAS 500) measured in the liver of HFD mice: **(A)** iNOS; **(B)** COX-2; **(C)** NF-κB; and **(D)** IκB-α expressions are reported as the ratio of optical densities of their bands to GAPDH. Immunoblots representative were shown. Densitometric evaluations of protein levels were reported. Data are expressed as means ± SEM (*n* = 6). *p < 0.05 and **p < 0.01, and ***p < 0.001 vs. STD; #*p* < 0.05 and ##*p* < 0.01 vs. HFD.

First, we evaluated the liver expression of pro- and anti-inflammatory enzymes. In vehicle-HFD mice, an increase in COX-2 and iNOS expressions were found (Figures 1A, B, black bars) with respect to vehicle-standard diet mice (white bars) (*p < 0.05 and **p < 0.01 vs. STD). PHAS treatment was able to reduce the expression of these inflammatory enzymes (#*p* < 0.05 vs. HFD). Moreover, accordingly, with these results, immunoreactivity for NF-κB was increased, whereas Iκb-α expression was significantly decreased in vehicle-HFD mice (***p < 0.001 and *p < 0.05 vs. STD, respectively), whereas PHAS treatment significantly restored their expressions (##*p* < 0.01 and #*p* < 0.05 vs. HFD, respectively) (Figures 1C, D, light blue vs. black bars).

**FIGURE 2:**
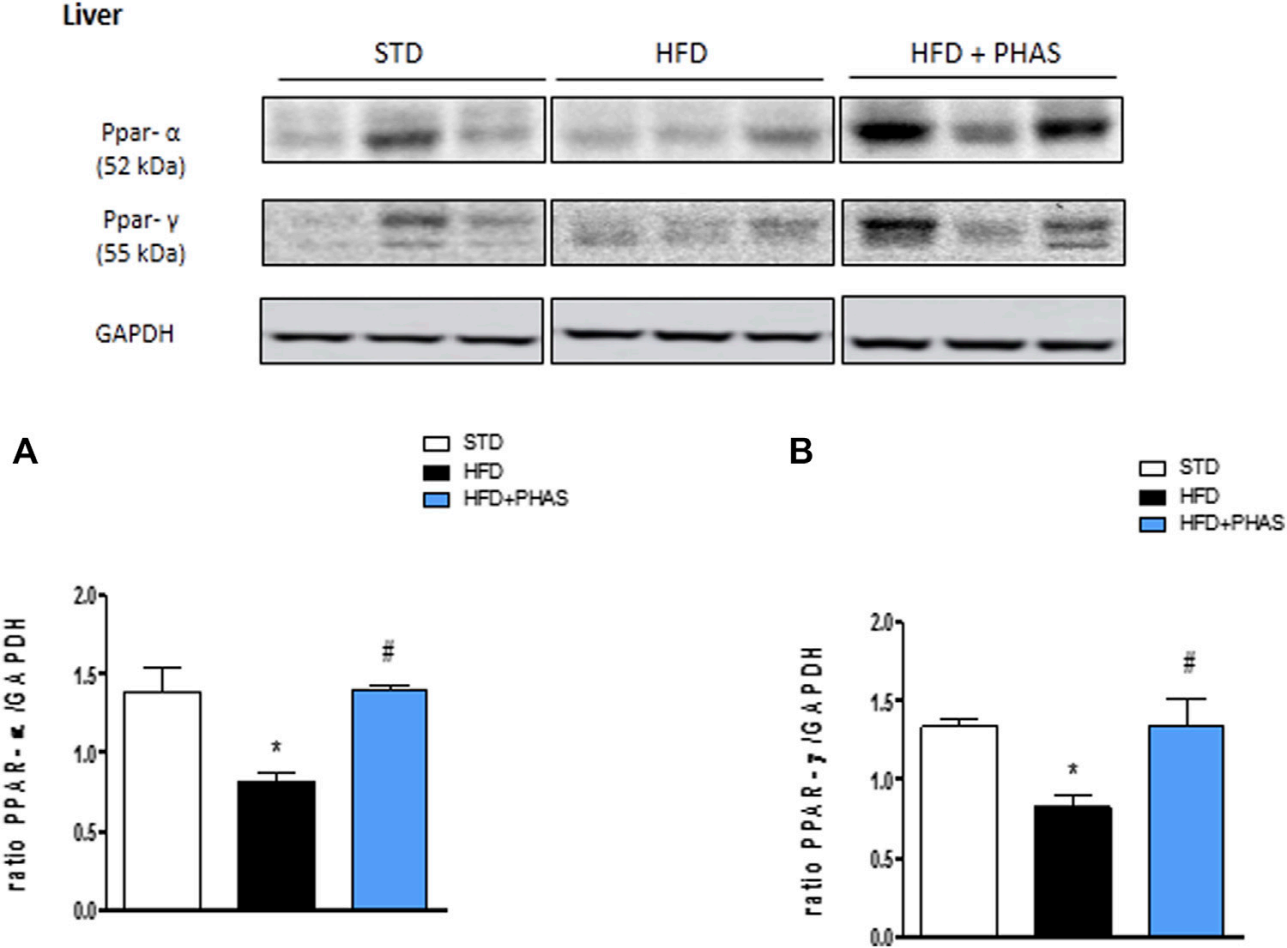
Effect of *P. vulgaris* extract (500 mg/kg, PHAS 500) on PPAR alpha **(A)** and gamma **(B)** and expressions in the liver of HFD mice. Levels are expressed as the density ratio of target to GAPDH. Data are expressed as means ± SEM (*n* = 6). *p < 0.05 vs. STD; #*p* < 0.05 vs. HFD.

In order to understand the protective effect by which PHAS may lower the development of HFD-induced liver inflammation, we evaluated the liver expressions of PPAR-α and PPAR-γ. Results showed that a significant reduction of both receptors was observed in vehicle-HFD mice (*p < 0.05 vs. STD) (Figures 2A, B, black vs. white bars), whereas PHAS treatment was able to restore their expressions (#*p* < 0.05 vs. HFD) (Figures 2A, B, light blue vs. black bars).

To strengthen the hypothesis that the PHAS anti-inflammatory activity is PPARs mediated, Spearman’s rank-order correlations were run to assess the relationship between different variables related to inflammation (namely, COX-2, iNOS, NF-κB, and Iκb-α) and PPAR-α and PPAR-γ, respectively, and regardless to diet (*n* = 9; three per group). When PPAR-γ and PPAR-α are related to inflammation, a negative correlation is expected with COX-2, iNOS, and NF-κB (pro-inflammatory) and a positive correlation with Iκb-α (anti-inflammatory).

As expected, a strong negative, statistically significant correlation was found between the PPAR-α and NF-κB (rs = −0.787, *p* = 0.012), whereas a strong positive correlation was found with Iκb-α (rs = 0.750, *p* = 0.020). Although not significant, moderate negative correlations were found with COX-2 (rs = −0.617, *p* = 0.077) and iNOS (rs = −0.650, *p* = 0.058).

Accordingly, strong negative, statistically significant correlations were found between COX-2 (rs = −0.717, *p* = 0.030), iNOS (rs = −0.700, *p* = 0.036), and PPAR-γ. Although not significante, a moderate negative correlation was found between NF-κB (rs = −0.644, *p* = 0.061) and PPAR-α, whereas a moderate positive correlation was found with Iκb-α (rs = 0.617, *p* = 0.077).

### GC-MS serum and fecal butyrate quantification

**FIGURE 3:**
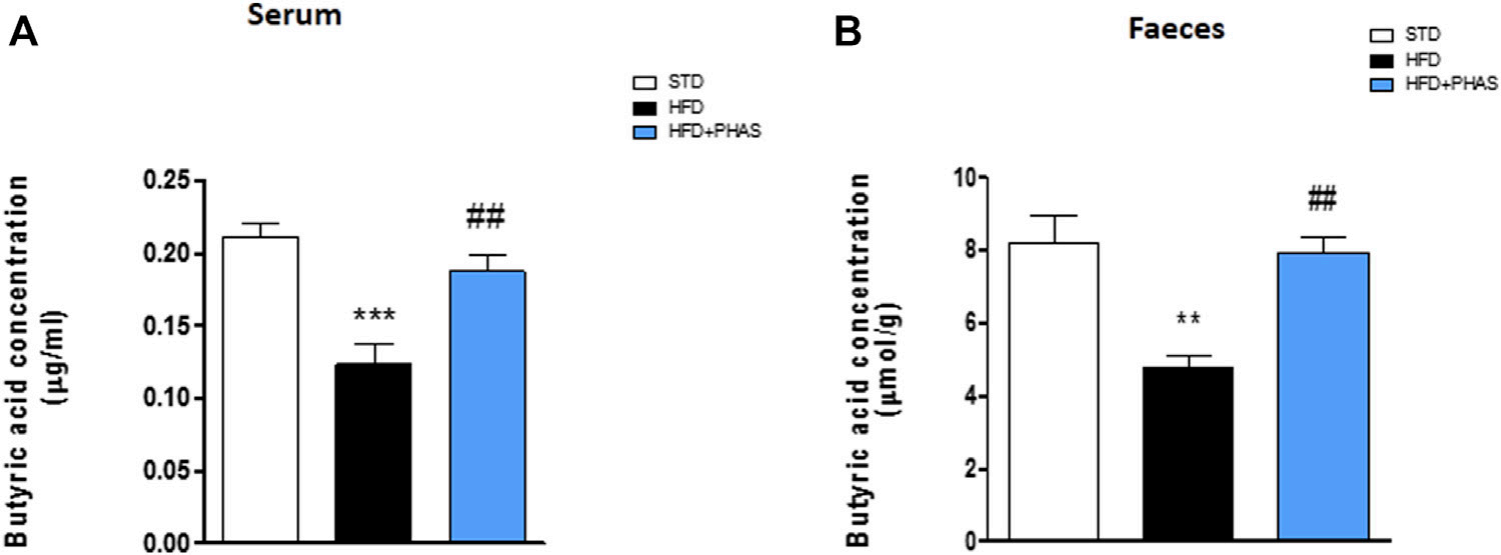
*P. vulgaris* extract effect on butyric acids levels in serum **(A)** and stools **(B)** both after 19 weeks from initiation of HFD diet, evaluated by GC-MS. Data are means ± SEM (*n* = 6). **p < 0.01 and ***p < 0.001 vs. STD; ##*p* < 0.01 vs. HFD.

Butyrate concentrations at the end of the experiment (week 19) were measured by GC-MS. Both fecal and serum butyrate values obtained from mice fed with a standard diet were significantly reduced in HDF mice (**p < 0.01 and ***p < 0.001 vs. STD) (Figures 3A, B, black vs. white bars). Surprisingly, butyric acid concentrations were significantly increased both in serum and in stool samples after PHAS treatment (##*p* < 0.01 vs. HFD) (Figures 3A, B, light blue vs. black bars).

### *Phaseolus vulgaris* extract treatment restored peroxisome proliferator-activated receptor expression and barrier integrity in the colon and cecum

**FIGURE 4:**
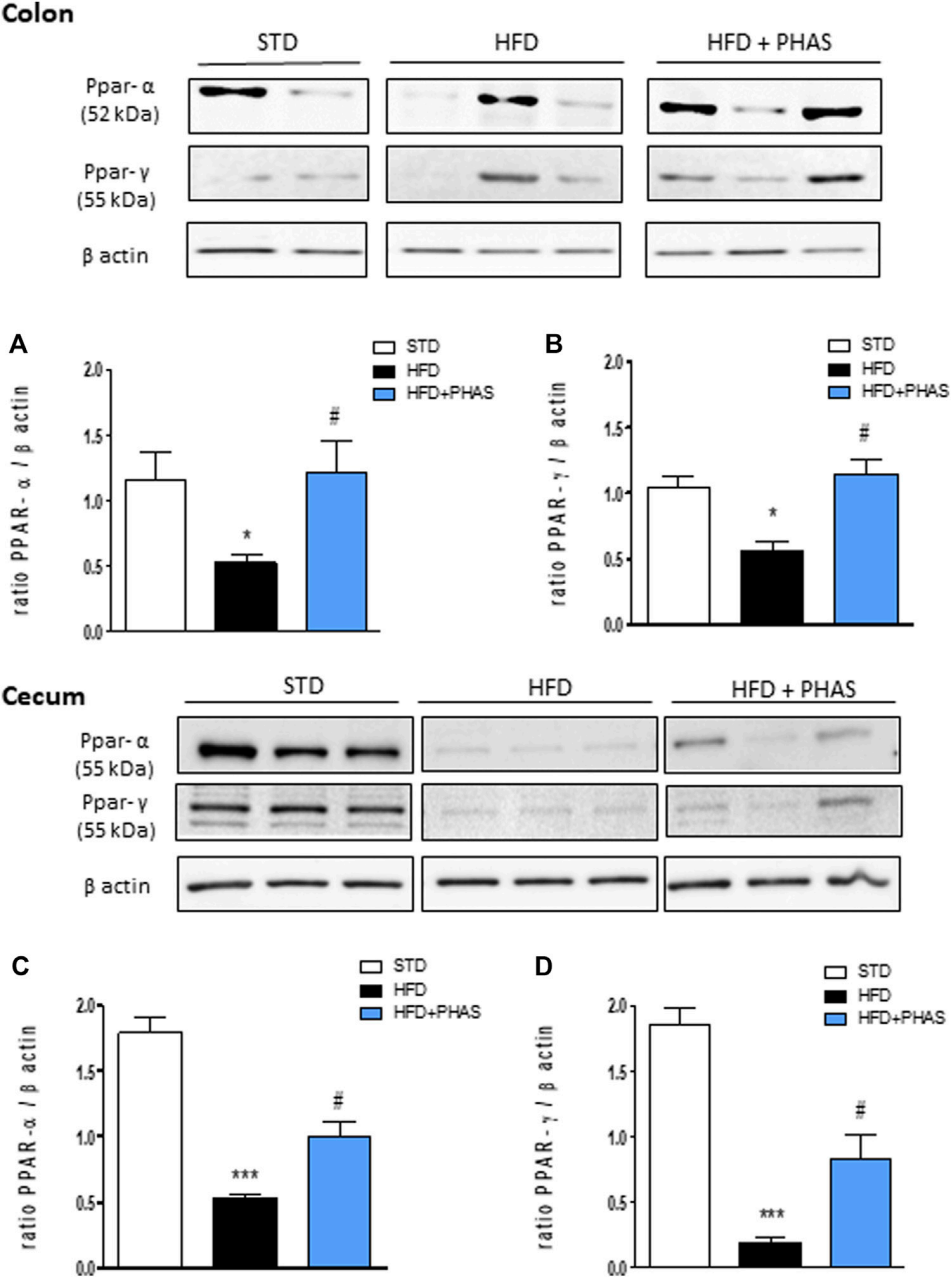
Effect of *P. vulgaris* extract (500 mg/kg, PHAS 500) on PPAR alpha **(A,C)** and gamma **(B,D)** and expressions in colon and cecum HFD mice, respectively, reported as the ratio of optical densities of their bands to β-actin. Immunoblot representatives were shown. Densitometric evaluations of protein levels were reported. Data are expressed as means ± SEM (*n* = 6). *p < 0.05 and ***p < 0.001 vs. STD; #*p* < 0.05 vs. HFD.

Then, we measured PPAR-α and γ expressions in the colon and cecum. As shown in Figure 4, HFD mice significantly decreased PPAR-α and PPAR-γ expression in the colon (*p < 0.05 and ***p < 0.001 vs. STD) (Figures 4A, B, black vs. white bars) and cecum (Figures 4C, D, black vs. white bars), and PHAS treatment blunted these effects (#*p* < 0.05 vs. HFD) (Figures 4C, D, light blue vs. black bars).

**FIGURE 5:**
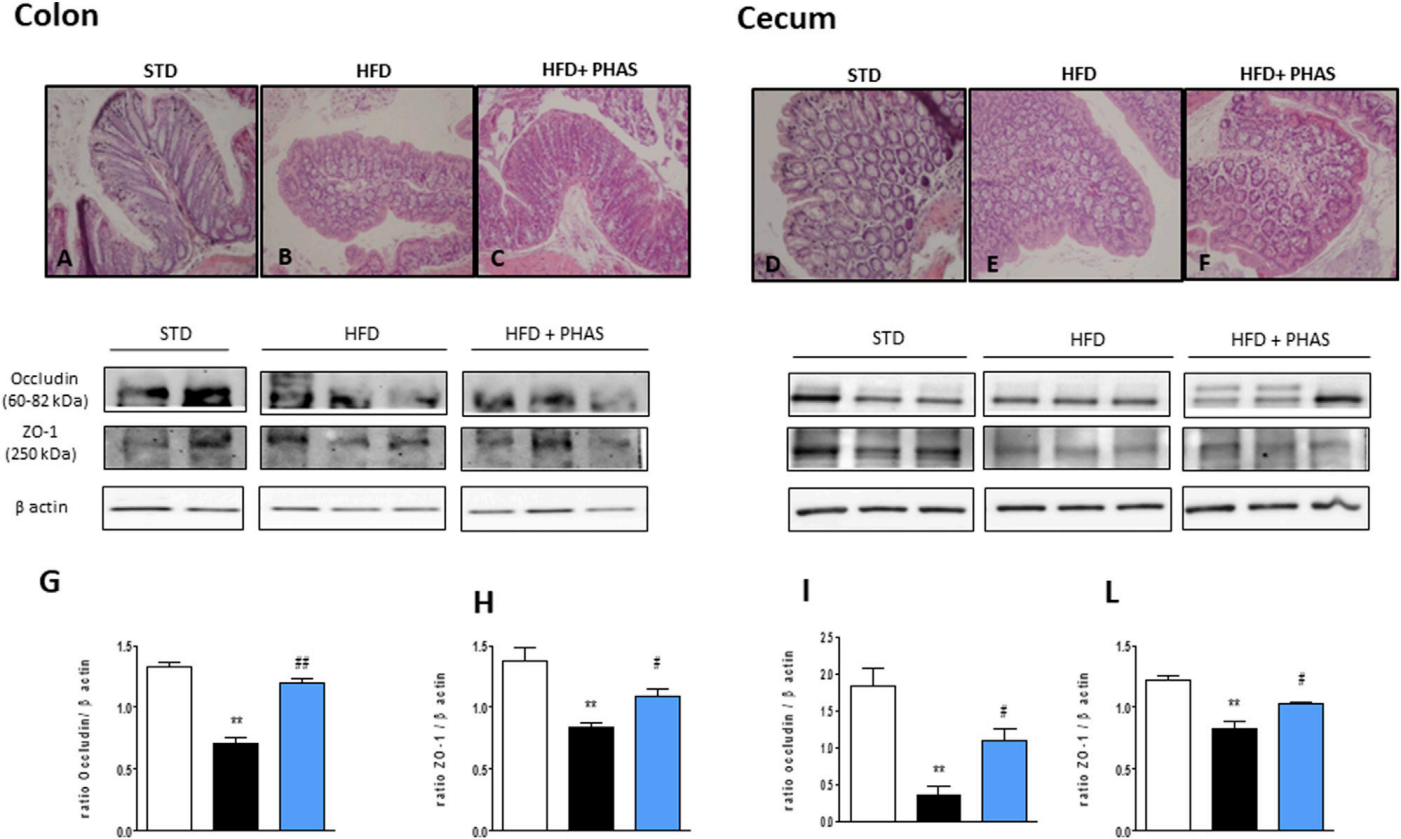
Histological changes of hematoxylin-eosin staining assay in colonic and cecum tissue in HFD mice induced by *P. vulgaris* extract (500 mg/kg, PHAS 500) treatment. Micrographs are representative pictures with an original magnification of ×20 (n = 4). **(A)** Colon tissue from the control group. **(B)** Colon tissue of HFD mice, showing severe goblet cell hyperplasia and mild epithelial hyperplasia with low-grade dysplasia. **(C)** Colon tissue of HFD + PHAS group, showing moderate goblet cell hyperplasia. **(D)** Cecum tissue from control mice. **(E)** Cecum tissue of HFD mice, showing severe goblet cell hyperplasia and mild epithelial hyperplasia with low-grade dysplasia. **(F)** Cecum tissue of HFD + PHAS group, showing moderate goblet cell hyperplasia. Protective effect of *P. vulgaris* extract (500 mg/kg, PHAS 500) treatment on colonic and cecum barrier integrity in HFD mice: in particular, immunoblot representatives of occludin expression in the colon **(G)** and cecum **(I)** and ZO-1 in the colon **(H)** and cecum **(L)** were showed. The levels are expressed as the density ratio of target to β-actin. Data are expressed as means ± SEM (*n* = 6). **p < 0.01 vs. STD; #*p* < 0.05 and ##*p* < 0.01 vs. HFD.

In order to understand if restored PPAR expression by PHAS treatment could influence intestinal homeostasis, histological evaluation of both the colon and cecum was conducted. Colon sections from HFD mice showed severe goblet cell hyperplasia and mild epithelial hyperplasia with low-grade dysplasia (Figure 5B), compared to normal diet + vehicle sections (STD, Figure 5A). PHAS treatment in injured mice ameliorated goblet cell hyperplasia (Figure 5C). Similarly, cecum sections were analyzed. HFD mice showed severe goblet cell hyperplasia and mild epithelial hyperplasia with low-grade dysplasia (Figure 5E) compared to STD diet mice (Figure 5D), while the HFD + PHAS group cecum showed moderate goblet cell hyperplasia (Figure 5F). Tight junction (TJ) status is a good marker for barrier integrity loss. Therefore, we investigated the expression of occludin and ZO-1, which are involved in preserving gut integrity (Odenwald and Turner, 2013). HFD mice showed a significant reduction of these tight junction proteins (**p < 0.01 vs. STD) (Figures 5G–L, black vs. white bars), and their level was restored by PHAS treatment (#*p* < 0.05 and ##*p* < 0.01 vs. HFD) (Figures 5G–L, light blue vs. black bars).

### Anti-inflammatory effect of *Phaseolus vulgaris* extract treatment in high-fat diet mice colon

**FIGURE 6:**
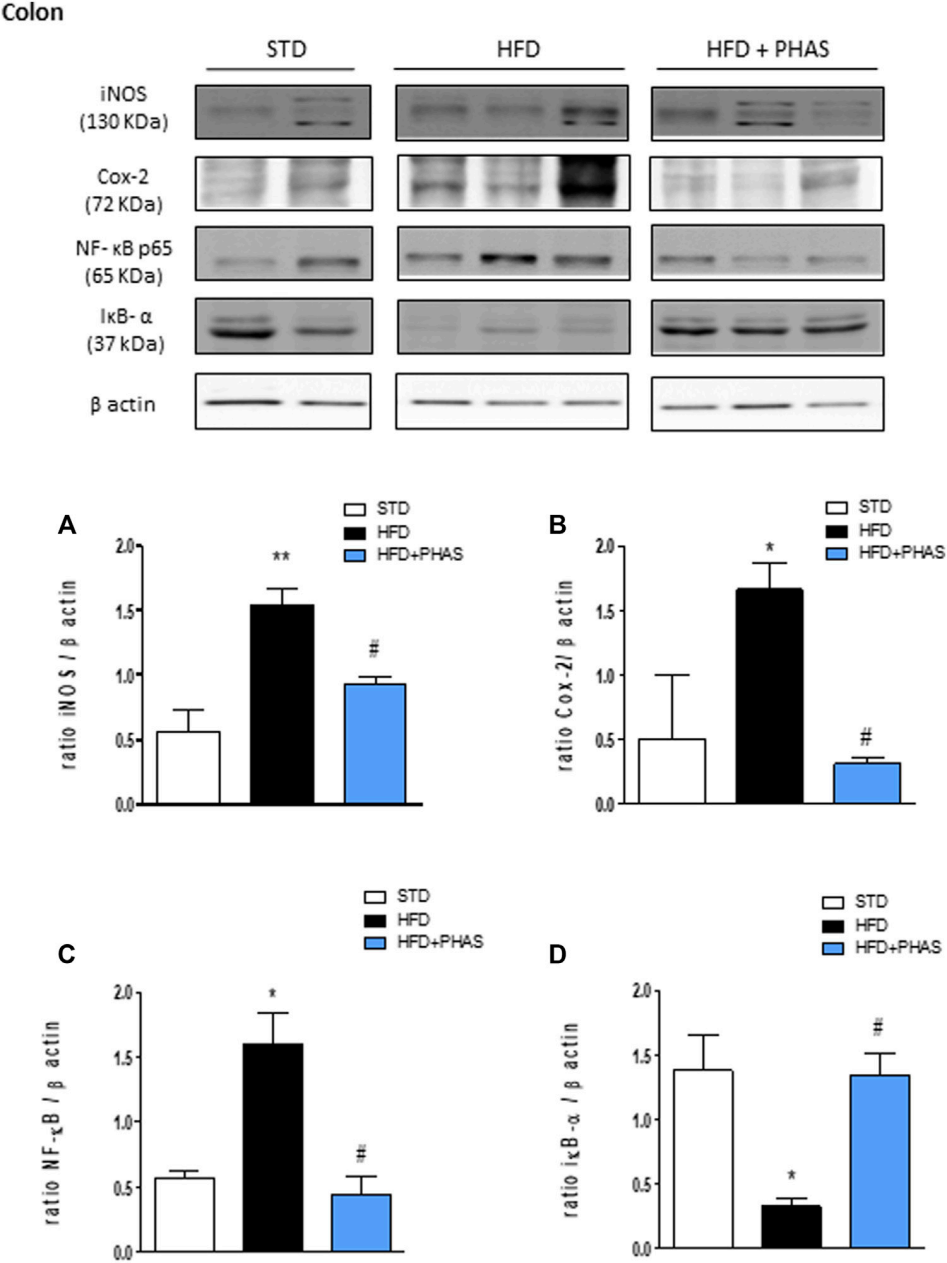
Anti-inflammatory effect of *P. vulgaris* extract (500 mg/kg, PHAS 500) measured using pro-inflammatory markers in the colon in HFD mice: **(A)** iNOS; **(B)** COX-2; **(C)** NF-κB; and **(D)** IκB-α expression levels are reported as the ratio of optical densities of their bands to β-actin. Immunoblot representatives were shown. Densitometric evaluations of protein levels were reported. Data are expressed as means ± SEM (*n* = 6). *p < 0.05 and **p < 0.01 vs. STD; #*p* < 0.05 vs. HFD.

Then, the expressions of pro- and anti-inflammatory enzymes in the colon were assessed. We considered iNOS and COX-2 expressions: both enzymes’ expressions were significantly increased in HFD mice (*p < 0.05 and **p < 0.01 vs. STD) (Figures 6A, B, black vs. white bars), while PHAS treatment was able to normalize their expression (#*p* < 0.05 vs. HFD) (Figures 6A, B, light blue vs. black bars). Furthermore, as these enzymes are induced by NF-κB complex activation, we also evaluated their expression, together with the cytosolic expression of the inhibitory protein of NF-κB and IκBα. HFD induced the nuclear translocation of the p65 subunit of NF-κB (Figure 6C, black vs. white bars) and decreased IκBα expression (*p < 0.05 vs. STD) (Figure 6D, black vs. white bars). These effects were completely prevented by PHAS treatment (#*p* < 0.05 vs. HFD) as there were no significant differences between HFD + PHAS-treated mice and controls (STD) (Figures 6C, D, light blue vs. black bars).

### *Phaseolus vulgaris* extract treatment prevents caecal inflammation induced by high-fat diet

**FIGURE 7:**
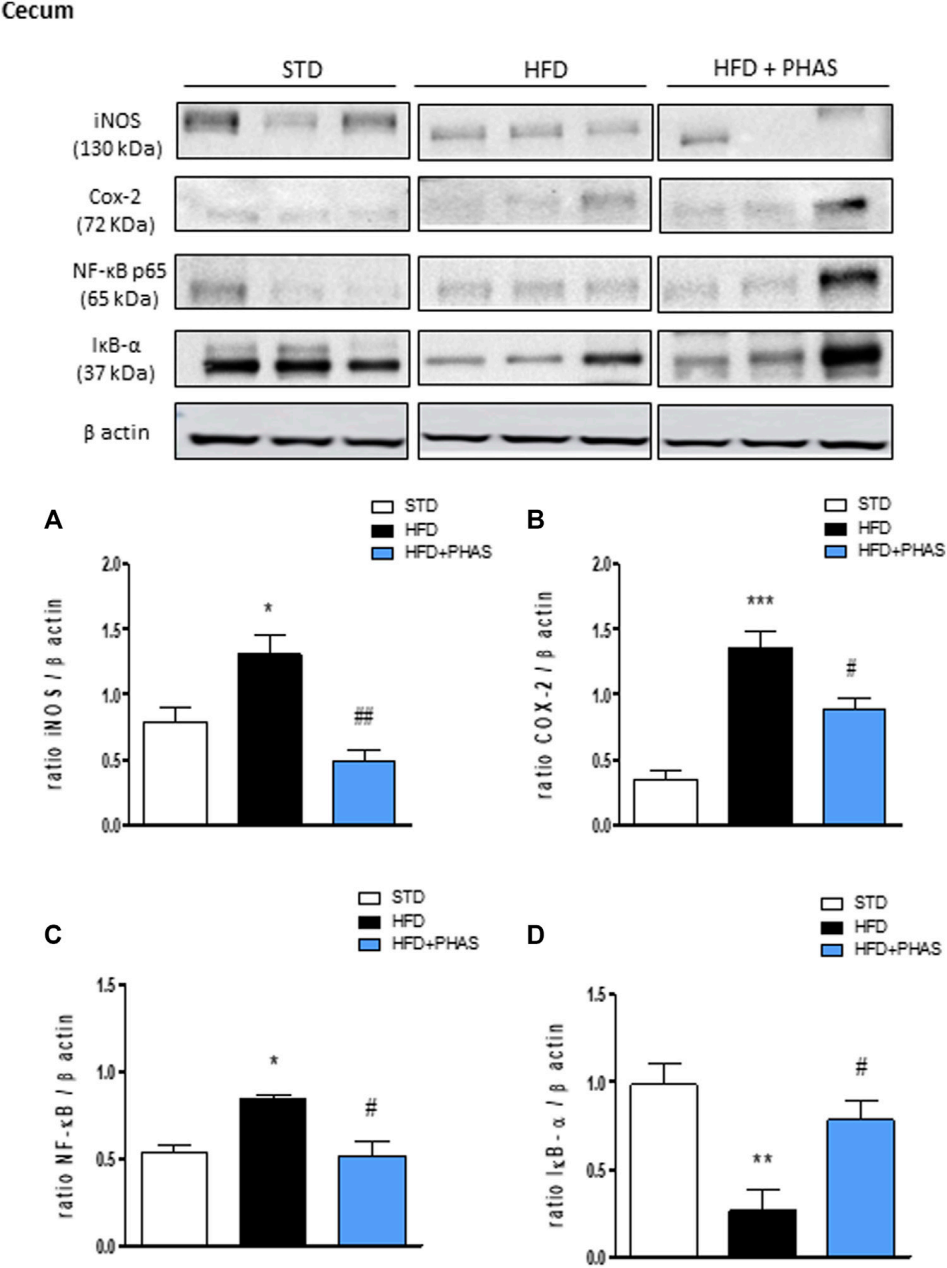
Effect of *P. vulgaris* extract (500 mg/kg, PHAS 500) treatment on **(A)** iNOS, **(B)** COX-2, **(C)** NF-κB, and **(D)** IκB-α in the cecum in HFD mice. Densitometric analysis of protein bands is reported: the levels are expressed as the density ratio of target to β-actin. Data are expressed as means ± SEM (*n* = 6). *p < 0.05 and **p < 0.01, and ***p < 0.001 vs. STD; #*p* < 0.05 and ##*p* < 0.01 vs. HFD.

Finally, the same evaluation was carried out also in the cecum. As pointed out in colon tissue, Western blot analysis showed that HFD significantly induced the expression of iNOS, COX-2, and the nuclear translocation of the p65 subunit of NF-κB and decreased the cytosolic expression of IκBα (*p < 0.05, **p < 0.01, and ***p < 0.001 vs. STD) (Figures 7A–D, black vs. white bars). All these effects were completely prevented by PHAS treatment (Figures 7A–D, light blue vs. black bars) in a significant manner (#*p* < 0.05 and ##*p* < 0.01 vs. HFD).

## Discussion

In this study, we demonstrate that a chronic oral PHAS treatment ameliorates some of the features related to obesity in HFD-induced obese mice. We investigate the mechanisms underpinning liver and intestinal inflammation, focusing on the role of PPARs and butyrate levels in serum and feces in this scenario.

The liver plays a key role in metabolic homeostasis, predisposing the whole body to inflammation when metabolism is compromised. Inflammation is largely considered the driving force for the progression or exacerbation of metabolic diseases, such as dyslipidemia, insulin resistance, and hepatic steatosis. The onset of metabolic syndrome was induced by HFD, containing 60% fat out of total calories, composed of animal fat and sucrose, as previously reported (Micheli et al., 2019). Previous pieces of evidence have pointed out that HFD induced a low-grade chronic inflammation (Kim et al., 2012) and an impairment of gut barrier function (De La Serre et al., 2010) when compared to a standard diet. In addition, HFD led to hepatic inflammation and oxidative stress (Pirozzi et al., 2016).

Herein, we showed that PHAS extract treatment significantly ameliorates liver inflammation induced by HFD. Since PPAR-α and γ can modulate metabolic disorders associated with inflammation (Pirozzi et al., 2016) and the inflammatory process itself (D’Agostino et al., 2007; Bensinger and Tontonoz, 2008; Russo et al., 2016), we evaluated if PHAS treatment is able to influence PPAR expression. For the first time, we show that the anti-inflammatory activity of PHAS is strictly correlated to PPAR-α and γ expression restoration in the liver.

Recently, it has been reported that obesity is linked to many other deleterious downstream effects, such as chronic intestinal inflammation and gut microbial dysbiosis, with a reduction of many butyrate-producing bacteria (Noureldein et al., 2020). Indirectly, this situation can lead to a reduction in circulating and fecal butyrate levels (Cani et al., 2008). About that, we demonstrate that PHAS treatment was able to restore butyrate levels in a significant manner, both in serum and in feces. This effect could be due to the inhibition of the absorption of starch in the gastrointestinal tract, which induces an increased colonic fermentation by the gut microbiota, as previously reported (Shi et al., 2020). Butyrate has a vast beneficial effect in energy metabolism, intestinal homeostasis, and immune response regulation. In particular, butyrate might have the potential in alleviating obesity and related comorbidities (Coppola et al., 2021). It has also been reported that butyrate is able to upregulate hepatic expression of PPAR-α, an essential regulator for mitochondrial fatty acid oxidation, alleviating HFD-induced NAFLD in rats (Sun et al., 2018). Therefore, we hypothesized that PHAS treatment increases butyrate circulating and fecal levels, probably influencing butyrate-bacteria species production, and in turn, butyrate upregulates PPAR expressions in the liver.

Moreover, it was reported that HFD could cause steatohepatitis but also a gut microbiota alteration together with a disruption of the intestinal barrier (Cani et al., 2007; Kim et al., 2012). In this scenario, gut microbiota plays a key role in various physiological and pathological processes through regulating short-chain fatty acid production, bile acids, and amino acids, and it is responsible for the development of metabolic syndromes like obesity and diabetes; however, the pathogenesis is not fully clear (Cani et al., 2008). In addition, it is clear that gut microbiota affects nuclear receptors, such as PPARs (Mirza et al., 2019). PPARs play an important role in the host-gut microbiome crosstalk, and they have been identified as enteric epithelial homeostasis mediators (Gao et al., 2018). Our results showed that these nuclear receptors are significantly downregulated in HFD mice, both in the colon and in the cecum, if compared to mice fed with a standard diet. Even in this case, PHAS treatment is able to restore their expression. Another interesting question that was recently pointed out is that bacteria and gut-derived products can communicate not only with neighboring but also with distant organs and tissues in the body, such as the brain (by gut–brain axis), liver (by gut–liver axis), and immune system (Schroeder and Bäckhed, 2016). TJ proteins and mucus secreted by intestinal epithelial cells play an important role in preventing intestinal flora translocation (Bu et al., 2020). In fact, intestinal mucosal barrier dysfunction, including increased mucosal permeability, damage to intestinal TJ proteins, sparse shedding of intestinal villi, increased pathogenic bacteria, and imbalance of intestinal flora, might be an important mechanism of hyperlipidemia (Wigg et al., 2001). Micheli and co-workers have already demonstrated that PHAS has an anti-hyperlipidemic activity, so we have analyzed intestinal barrier integrity, both at colon and cecum levels. In particular, PHAS treatment is able to restore occludin and ZO-1 expression in a significant manner in mice colonic tissue and to ameliorate histological features, such as epithelial and goblet cell hyperplasia, together with dysplasia. Brahe and co-workers suggested that an increased level of butyrate-producing bacteria in the intestinal microbiota might alleviate obesity and related metabolic complications based on the potential anti-inflammatory and intestinal barrier function benefits of butyrate (Brahe et al., 2013). In fact, finally, we evaluated pro- and anti-inflammatory proteins both in the colon and in the cecum. Our results showed that PHAS treatment was able to restore the intestinal inflammatory balance that was disturbed in HFD mice. As well known, perturbated homeostasis can promote inflammation not only in the gastrointestinal tract but also in the liver by gut–liver axis, which can activate a cascade of severe events, leading to insulin resistance, liver inflammation, and fibrosis (Wang et al., 2017).

In summary, our data clearly demonstrate that a standardized *P. vulgaris* extract (PHAS) treatment protects the liver from damage induced by HFD. This effect was mediated by a reduction in hepatic inflammation by PPAR-α and γ expression restoration. Moreover, PHAS counteracts HFD-induced alteration of gut integrity, underpinned by the intestinal barrier integrity loss, and reduces systemic and fecal levels of butyrate, probably due to a decrease in butyrate-producing microbiota.

## Conclusion

PHAS treatment ameliorates liver and intestinal (colonic and caecal) dysfunction in adult obese mice, magnifying the cross talk between the liver and gut. Therefore, our data point out that PHAS can represent an additional tool for reducing some pathological features related to metabolic syndrome-like conditions induced by obesity, such as inflammation and intestinal barrier disruption.

## Data Availability

The original contributions presented in the study are included in the article/Supplementary Material; further inquiries can be directed to the corresponding author.
