# Supplementary material for: Phaseolus vulgaris extract ameliorates high-fat diet-induced colonic barrier dysfunction and inflammation in mice by regulating peroxisome proliferator-activated receptor expression and butyrate levels
Source: Front Pharmacol. 2022 Aug 11;13:930832. doi: 10.3389/fphar.2022.930832 (PMC9403263; doi:10.3389/fphar.2022.930832)
Supplement: Supplementary file 1 [file Presentation1.PPTX]

## Slide 1
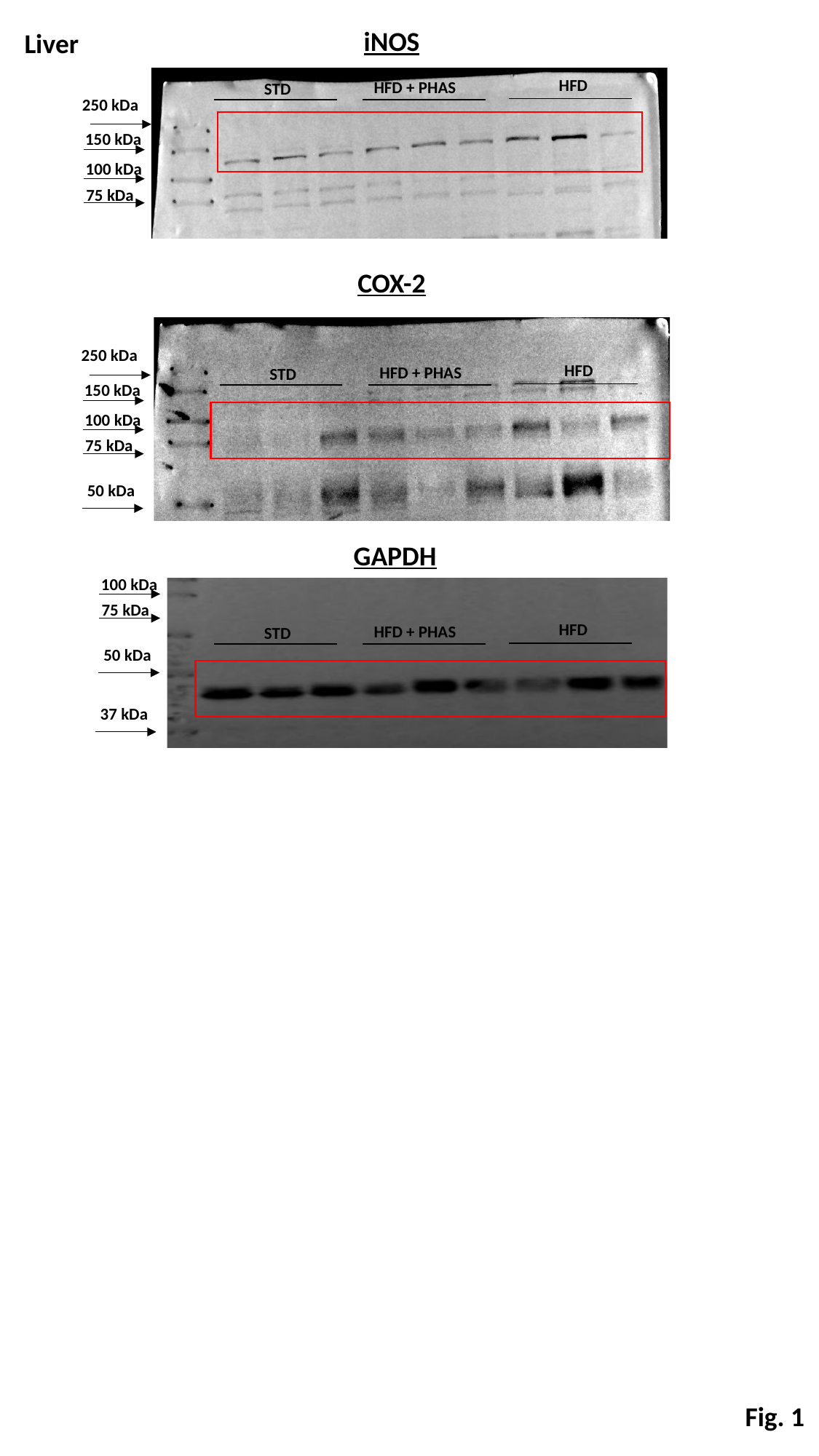

iNOS
Liver
HFD
HFD + PHAS
STD
250 kDa
150 kDa
100 kDa
75 kDa
COX-2
250 kDa
HFD
HFD + PHAS
STD
150 kDa
100 kDa
75 kDa
50 kDa
GAPDH
100 kDa
75 kDa
HFD
HFD + PHAS
STD
50 kDa
37 kDa
Fig. 1

## Slide 2
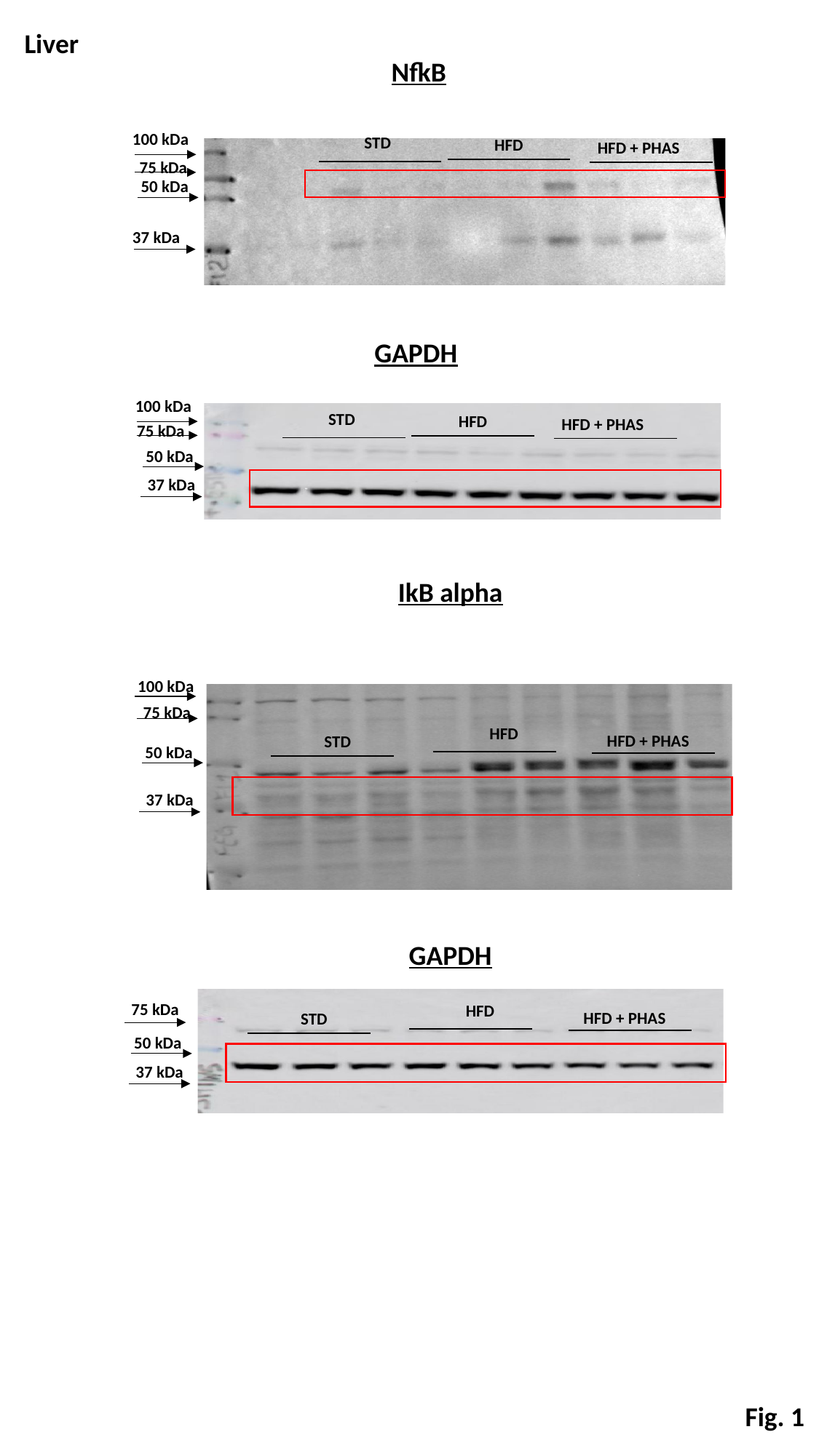

Liver
NfkB
100 kDa
STD
HFD
HFD + PHAS
75 kDa
50 kDa
37 kDa
GAPDH
100 kDa
STD
HFD
HFD + PHAS
75 kDa
50 kDa
37 kDa
IkB alpha
100 kDa
75 kDa
HFD
HFD + PHAS
STD
50 kDa
37 kDa
GAPDH
75 kDa
HFD
HFD + PHAS
STD
50 kDa
37 kDa
Fig. 1

## Slide 3
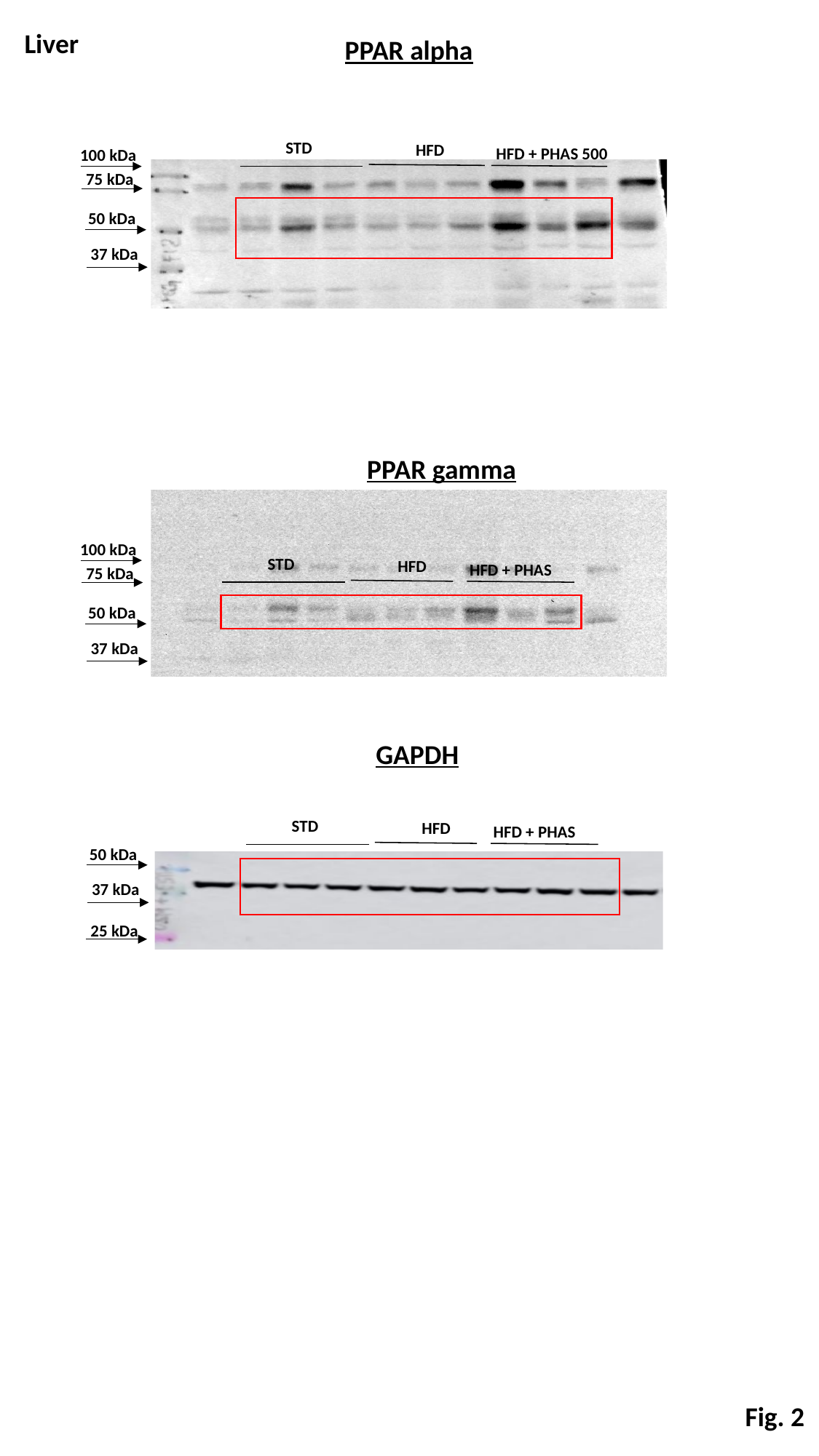

Liver
PPAR alpha
STD
HFD
HFD + PHAS 500
100 kDa
75 kDa
50 kDa
37 kDa
PPAR gamma
100 kDa
STD
HFD
HFD + PHAS
75 kDa
50 kDa
37 kDa
GAPDH
STD
HFD
HFD + PHAS
50 kDa
37 kDa
25 kDa
Fig. 2

## Slide 4
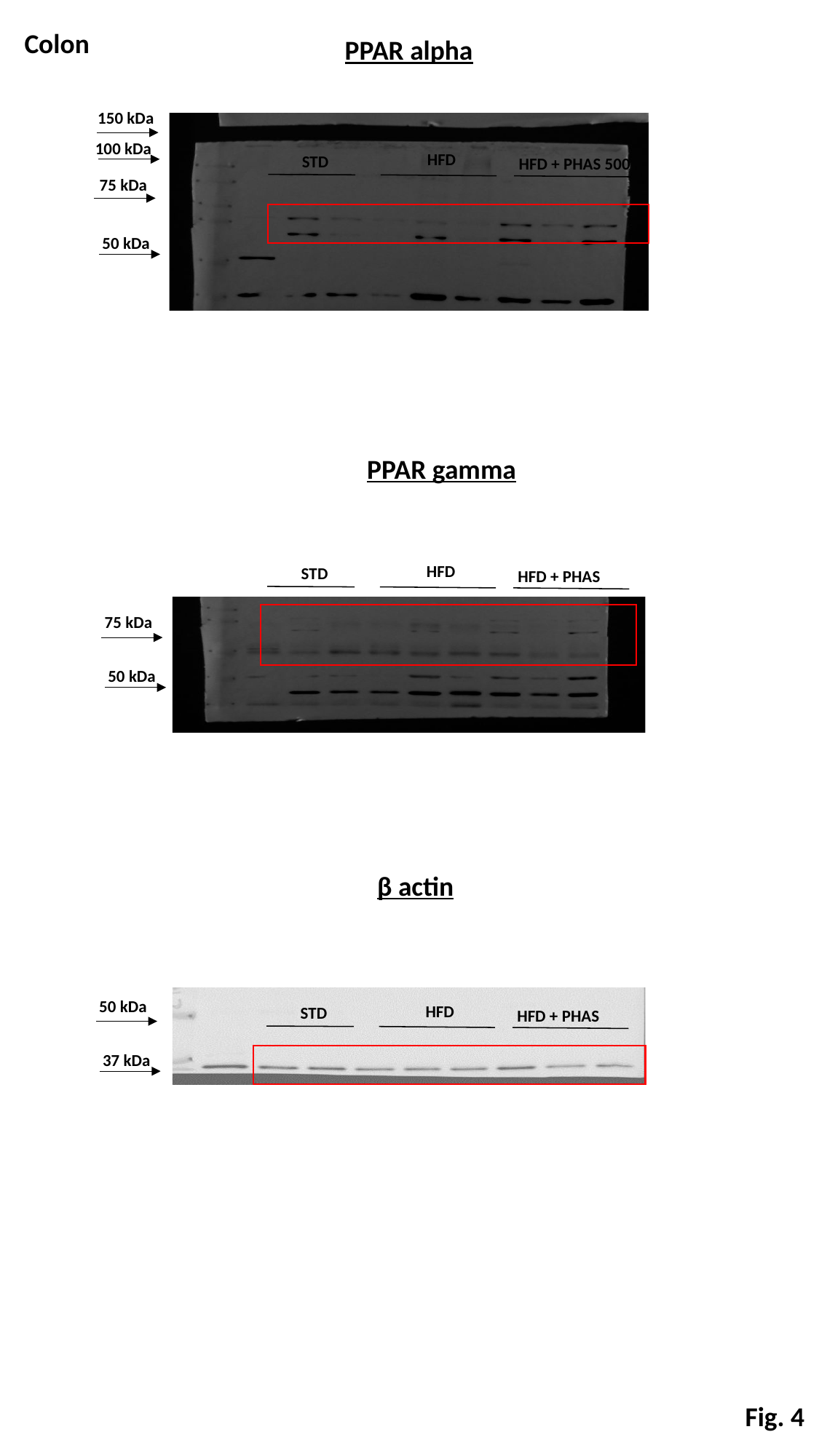

Colon
PPAR alpha
150 kDa
100 kDa
HFD
STD
HFD + PHAS 500
75 kDa
50 kDa
PPAR gamma
HFD
STD
HFD + PHAS
75 kDa
50 kDa
β actin
50 kDa
HFD
STD
HFD + PHAS
37 kDa
Fig. 4

## Slide 5
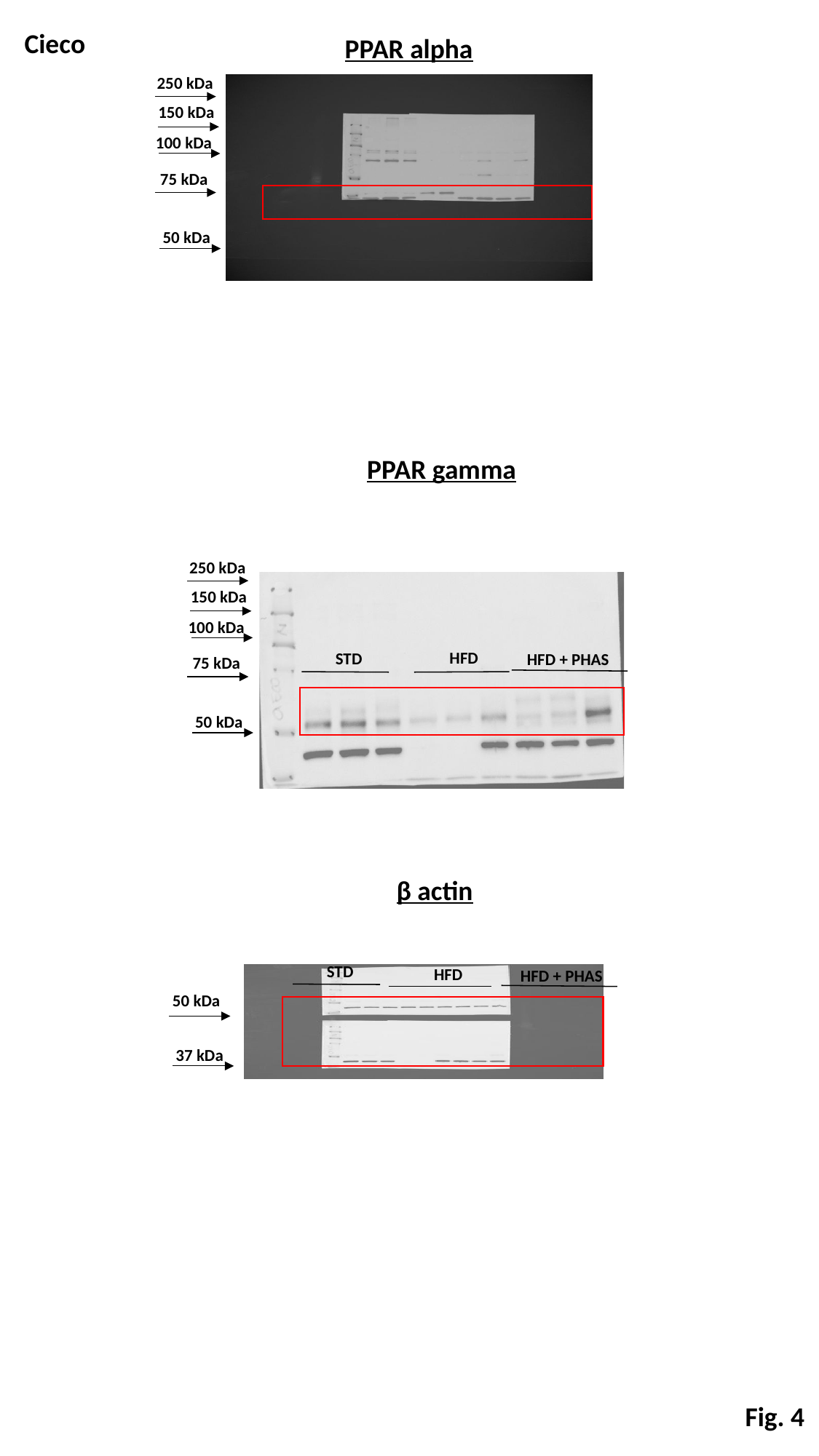

Cieco
PPAR alpha
250 kDa
150 kDa
100 kDa
75 kDa
50 kDa
PPAR gamma
250 kDa
150 kDa
100 kDa
HFD
STD
HFD + PHAS
75 kDa
50 kDa
β actin
STD
HFD
HFD + PHAS
50 kDa
37 kDa
Fig. 4

## Slide 6
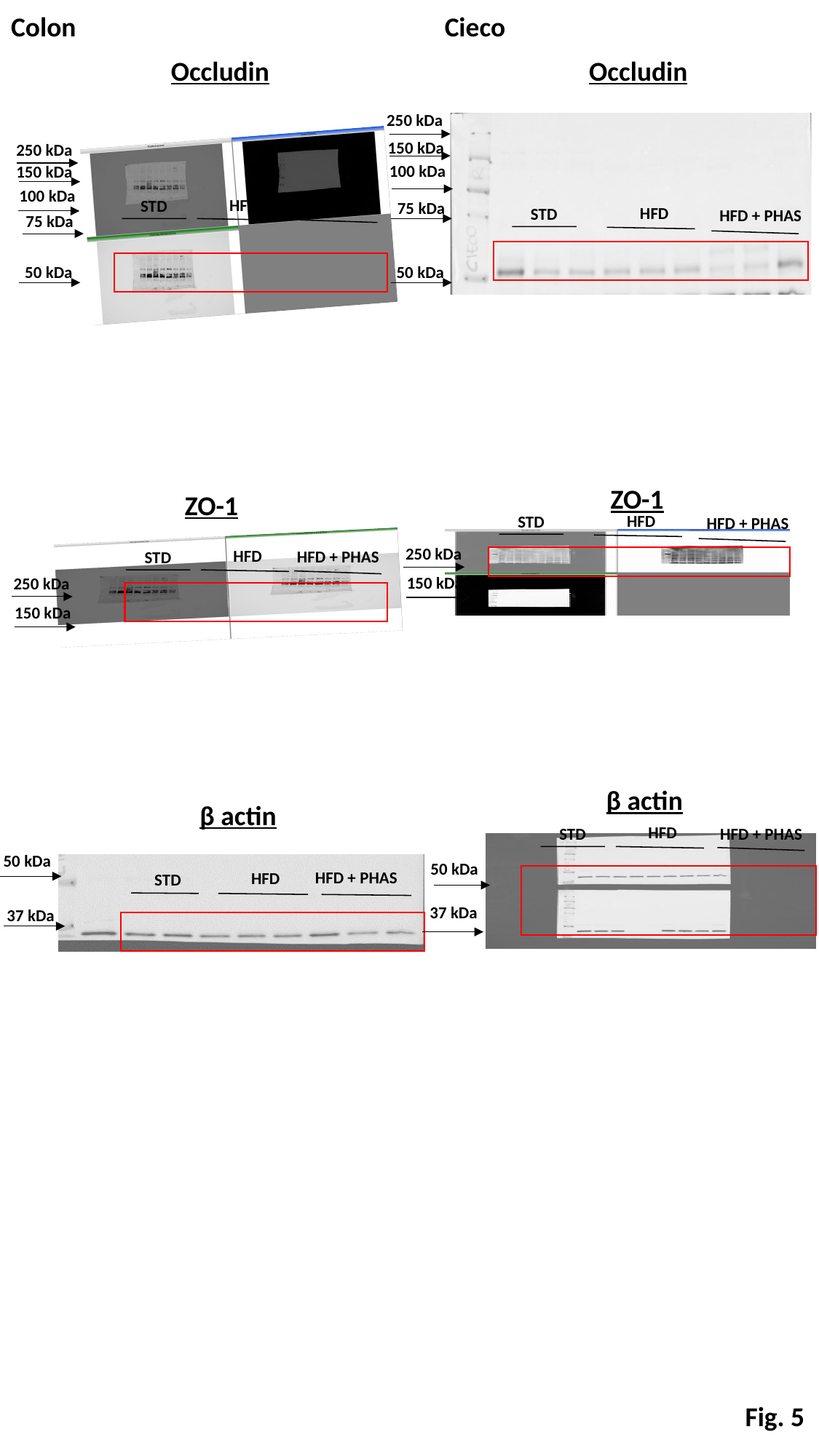

Colon
Cieco
Occludin
Occludin
250 kDa
150 kDa
250 kDa
100 kDa
150 kDa
100 kDa
HFD
HFD + PHAS
STD
75 kDa
HFD
STD
HFD + PHAS
75 kDa
50 kDa
50 kDa
ZO-1
ZO-1
HFD
STD
HFD + PHAS
250 kDa
HFD
HFD + PHAS
STD
150 kDa
250 kDa
150 kDa
β actin
β actin
HFD
HFD + PHAS
STD
50 kDa
37 kDa
50 kDa
HFD + PHAS
HFD
STD
37 kDa
Fig. 5

## Slide 7
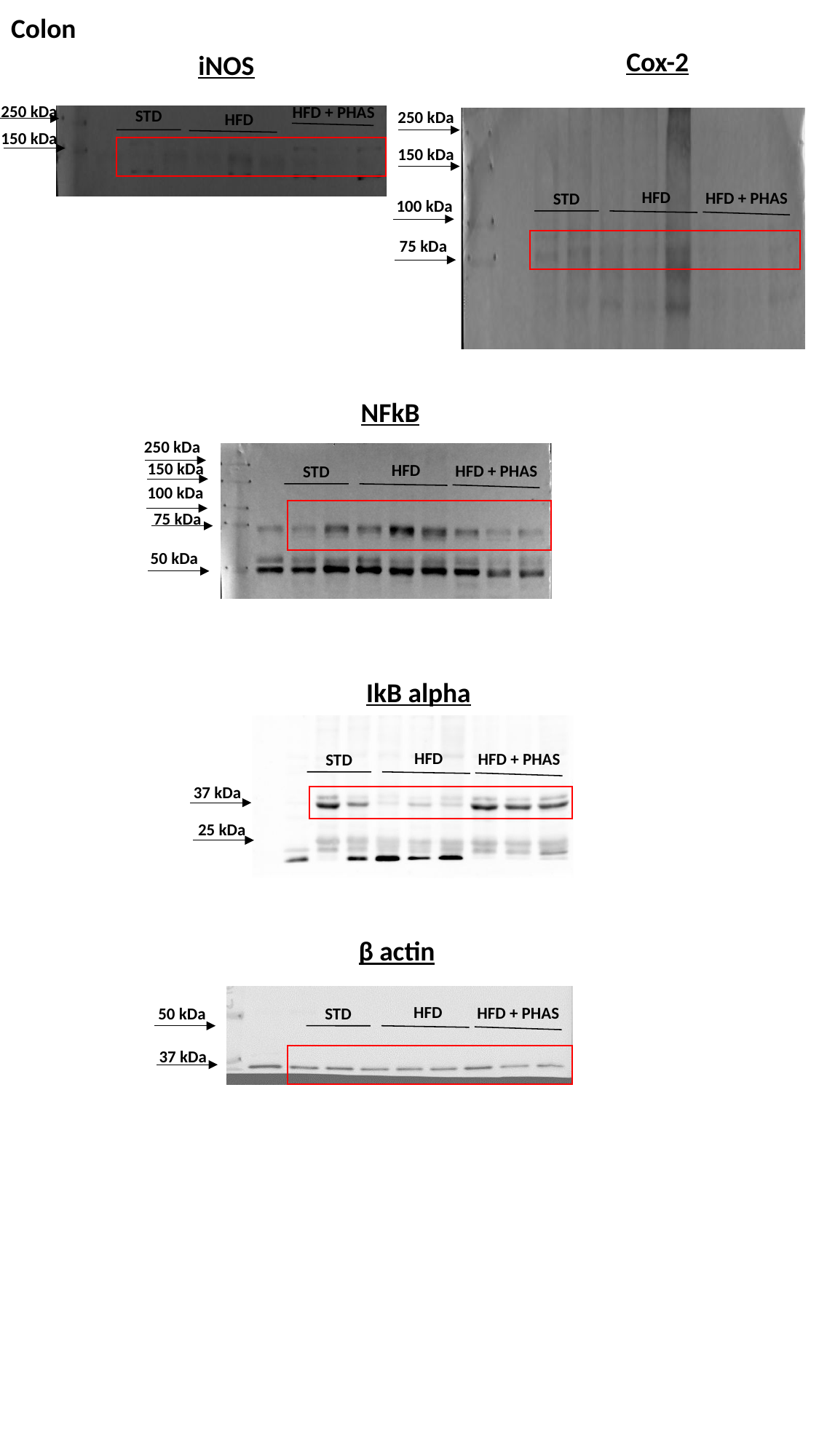

Colon
Cox-2
iNOS
250 kDa
HFD + PHAS
STD
250 kDa
HFD
150 kDa
150 kDa
HFD
HFD + PHAS
STD
100 kDa
75 kDa
NFkB
250 kDa
150 kDa
HFD
HFD + PHAS
STD
100 kDa
75 kDa
50 kDa
IkB alpha
HFD
HFD + PHAS
STD
37 kDa
25 kDa
β actin
HFD
HFD + PHAS
STD
50 kDa
37 kDa

## Slide 8
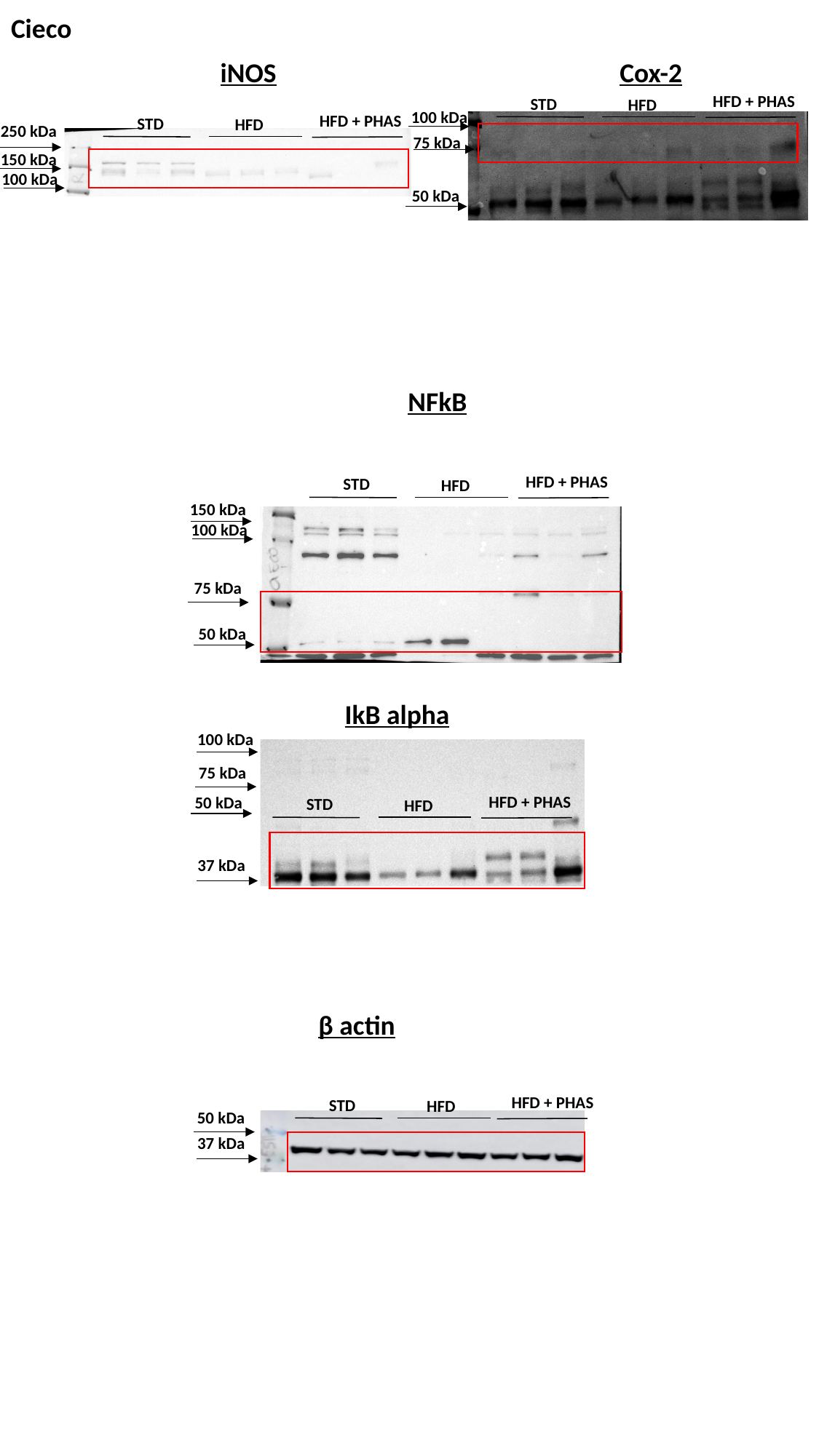

Cieco
iNOS
Cox-2
HFD + PHAS
STD
HFD
100 kDa
HFD + PHAS
STD
HFD
250 kDa
75 kDa
150 kDa
100 kDa
50 kDa
NFkB
HFD + PHAS
STD
HFD
150 kDa
100 kDa
75 kDa
50 kDa
IkB alpha
100 kDa
75 kDa
HFD + PHAS
50 kDa
STD
HFD
37 kDa
β actin
HFD + PHAS
STD
HFD
50 kDa
37 kDa
