# Supplementary figures and images for: Phaseolus vulgaris extract ameliorates high-fat diet-induced colonic barrier dysfunction and inflammation in mice by regulating peroxisome proliferator-activated receptor expression and butyrate levels
Source: Front Pharmacol. 2022 Aug 11;13:930832. doi: 10.3389/fphar.2022.930832 (PMC9403263; doi:10.3389/fphar.2022.930832)

## HFD

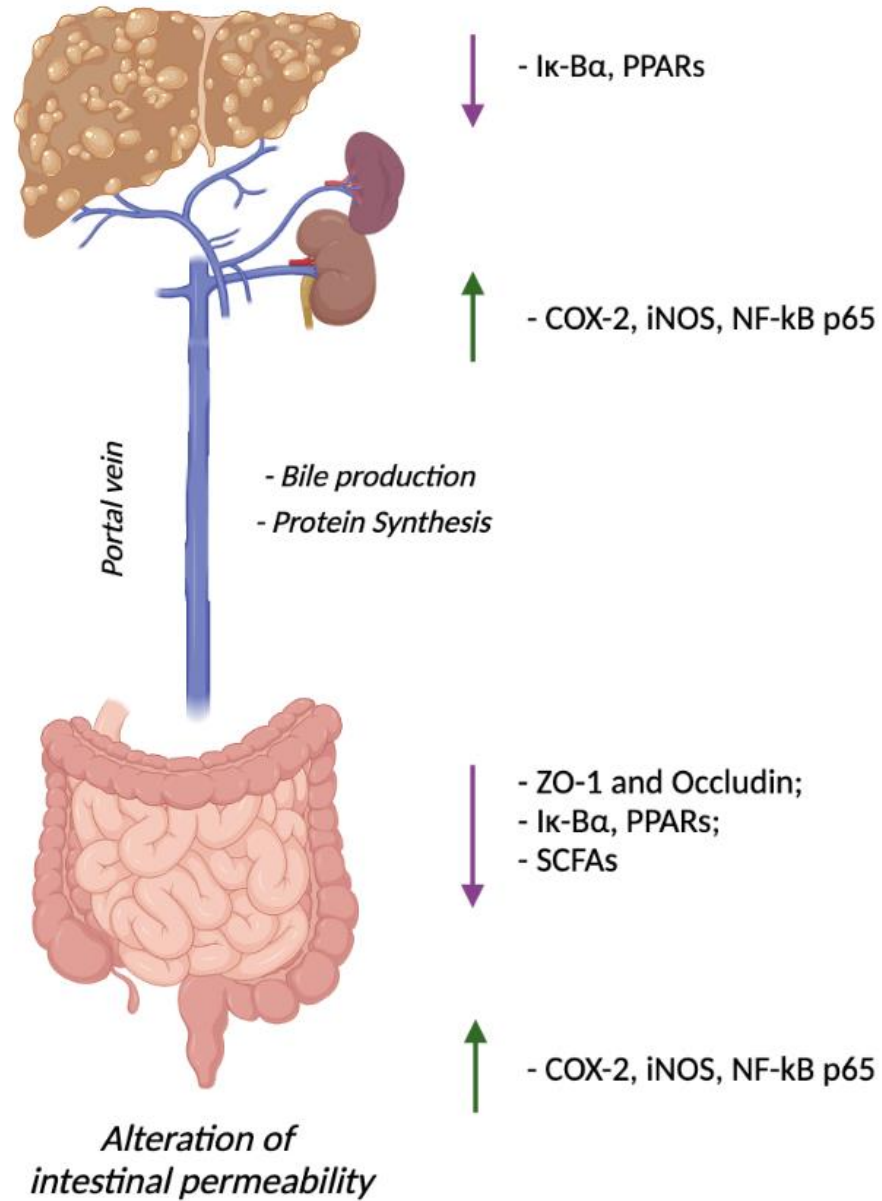

## PHAS TREATMENT

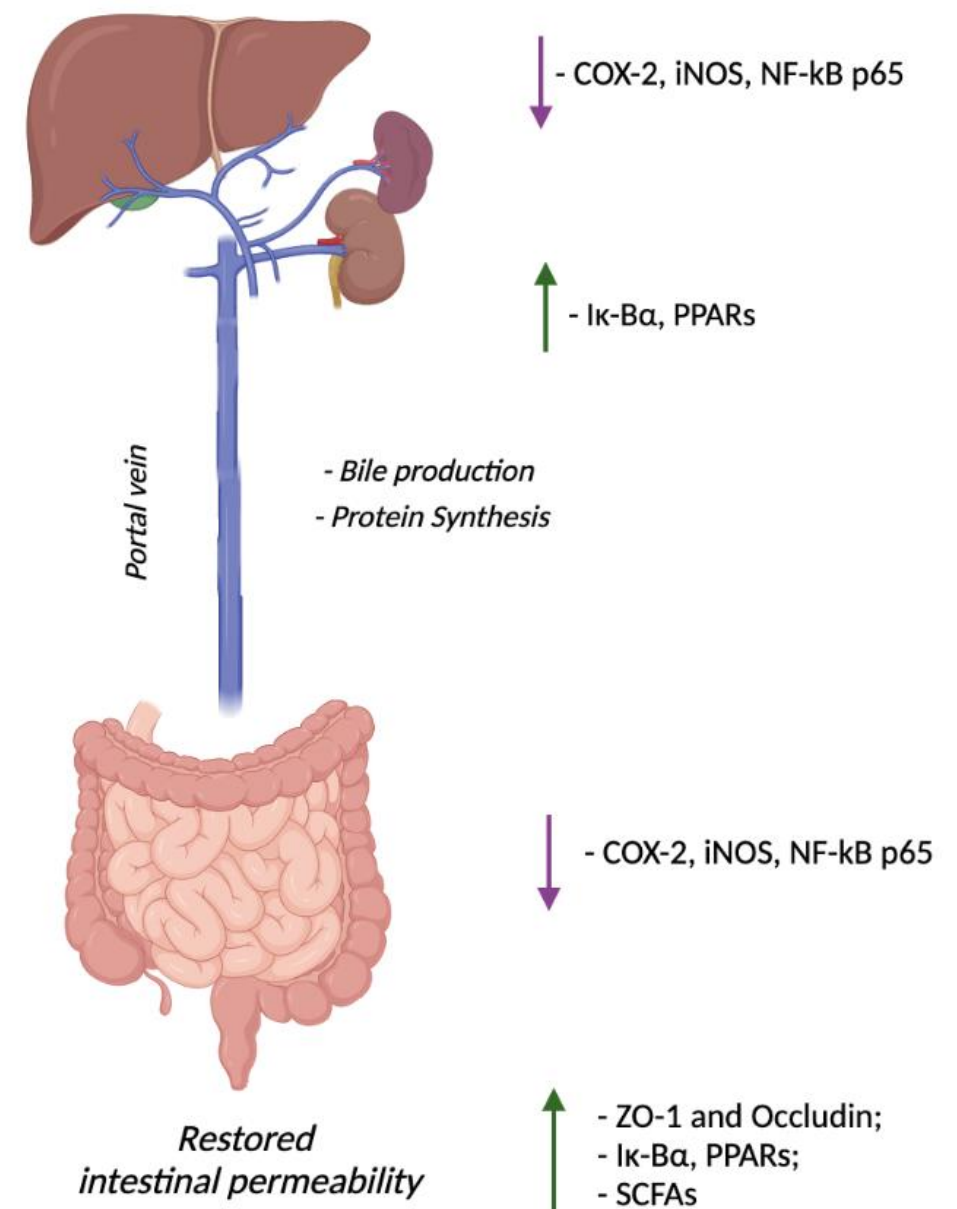

Supplement: Supplementary file 2 [file DataSheet1.PDF]
